# Adipokine Profile in Non-alcoholic Fatty Liver Disease

**DOI:** 10.7759/cureus.112017

**Published:** 2026-07-03

**Authors:** Trisha C, Prabhat Kumar N, Naveen Ch, Muralidhar Chinnapaka, Ramachakra Kushal Thota

**Affiliations:** 1 Department of Internal Medicine, Chalmeda AnandRao Institute of Medical Sciences, Karimnagar, IND; 2 Department of Internal Medicine, NIMRA Institute of Medical Sciences, Vijayawada, IND; 3 Department of General Surgery, Dr. Pinnamaneni Siddhartha Institute of Medical Sciences, Vijayawada, IND; 4 Department of Pharmacology, Government Medical College and General Hospital, Maheshwaram, Hyderabad, IND; 5 Department of Internal Medicine, Gandhi Medical College, Secunderabad, IND

**Keywords:** adipokines, adiponectin, dyslipidaemia, insulin resistance, leptin, non-alcoholic fatty liver disease, resistin, visfatin

## Abstract

Background: Non-alcoholic fatty liver disease (NAFLD) is commonly linked with metabolic disturbances such as central obesity, insulin resistance, dyslipidaemia and low-grade inflammation. Adipokines released from adipose tissue may influence hepatic fat accumulation, metabolic dysfunction and progression of liver injury. This study was conducted to assess the serum adipokine profile in patients with NAFLD, compare it with healthy controls and evaluate its relationship with anthropometric and biochemical parameters.

Materials and methods: This case-control study included 100 participants, consisting of 50 patients with ultrasound-diagnosed NAFLD and 50 age- and sex-matched healthy controls. Demographic details, body mass index, waist circumference, fasting blood glucose, fasting insulin, lipid profile and liver function parameters were recorded. Serum adiponectin, leptin, resistin and visfatin levels were measured using enzyme-linked immunosorbent assay. Insulin resistance was assessed using the Homeostatic Model Assessment of Insulin Resistance (HOMA-IR) index. The data were analysed to compare adipokine levels between the two groups and to determine their association with metabolic variables.

Results: The mean age of patients with NAFLD was 43.8 ± 9.6 years. Among them, 29 patients (58.0%) were males and 21 patients (42.0%) were females. Compared with controls, patients with NAFLD had significantly higher body mass index (29.1 ± 3.8 vs 23.7 ± 2.9 kg/m²), waist circumference (96.4 ± 8.7 vs 82.6 ± 7.9 cm), fasting blood glucose (112.8 ± 18.5 vs 91.6 ± 10.7 mg/dL), triglycerides (186.5 ± 42.3 vs 124.7 ± 31.8 mg/dL) and HOMA-IR (3.8 ± 1.2 vs 1.7 ± 0.6), with p < 0.001 for all comparisons. Serum adiponectin was significantly lower in the NAFLD group than in controls (5.9 ± 2.1 vs 10.8 ± 3.4 µg/mL; p < 0.001). In contrast, serum leptin (24.6 ± 8.9 vs 12.3 ± 5.4 ng/mL), resistin (14.8 ± 4.6 vs 8.7 ± 3.1 ng/mL) and visfatin (32.5 ± 10.2 vs 18.9 ± 7.6 ng/mL) were significantly higher among patients with NAFLD (p < 0.001). Adiponectin showed a negative correlation with body mass index, triglycerides and HOMA-IR, whereas leptin and resistin showed positive correlations with these parameters.

Conclusion: Patients with NAFLD showed a clear adipokine imbalance, characterised by reduced adiponectin and increased leptin, resistin and visfatin levels. This pattern was associated with obesity, dyslipidaemia and insulin resistance, indicating that adipokine alterations may contribute to the metabolic and inflammatory mechanisms involved in NAFLD.

## Introduction

Non-alcoholic fatty liver disease (NAFLD), now increasingly discussed under the broader term metabolic dysfunction-associated steatotic liver disease (MASLD), is one of the most common causes of chronic liver disease worldwide [1,2]. It includes a wide clinical spectrum, beginning with simple hepatic steatosis and progressing in some patients to steatohepatitis, fibrosis, cirrhosis and hepatocellular carcinoma [2,3]. The recent change in terminology highlights the close relationship between fatty liver, obesity, insulin resistance, dyslipidaemia and other cardiometabolic risk factors, although the term NAFLD continues to be widely used in clinical studies and routine practice [1,2]. Current global evidence suggests that nearly one-third of adults may have NAFLD, with a substantially higher burden among individuals with obesity and type 2 diabetes mellitus [4,5].

NAFLD is no longer viewed only as a liver disorder. It is strongly linked with metabolic syndrome, cardiovascular disease, chronic kidney disease and systemic inflammation [2,6,7]. Insulin resistance plays a central role in its pathogenesis by increasing adipose tissue lipolysis and free fatty acid delivery to the liver. This promotes hepatic triglyceride accumulation, oxidative stress, mitochondrial dysfunction and hepatocellular injury [8,9]. However, fat accumulation alone does not fully explain why only some patients develop inflammation, fibrosis and progressive liver damage. This has led to increasing interest in adipose tissue-derived mediators, particularly adipokines, as important contributors to NAFLD development and progression [10,11].

Adipose tissue is an active endocrine organ that secretes several biologically active molecules, including adiponectin, leptin, resistin, visfatin and inflammatory cytokines [10,12]. These mediators regulate appetite, insulin sensitivity, lipid handling, glucose metabolism, endothelial function and inflammatory responses [11,12]. In obesity and metabolic syndrome, adipose tissue becomes dysfunctional and develops an altered secretory pattern. This imbalance usually involves reduced levels of protective adipokines and increased levels of pro-inflammatory adipokines, which may worsen hepatic steatosis and metabolic stress [12,13].

Adiponectin is considered one of the most important protective adipokines in NAFLD. It improves insulin sensitivity, enhances fatty acid oxidation and has anti-inflammatory and anti-fibrotic actions [13,14]. Lower adiponectin levels have been reported in patients with NAFLD and are often associated with obesity, insulin resistance and adverse lipid parameters [14,15]. In contrast, leptin levels are usually elevated in obesity and NAFLD. Although leptin has a physiological role in appetite control and energy balance, chronic hyperleptinaemia and leptin resistance may promote inflammation and hepatic fibrogenesis [16,17].

Resistin and visfatin are also being studied as possible markers of metabolic and inflammatory activity in NAFLD. Resistin has been associated with insulin resistance, endothelial dysfunction and inflammatory activation [18]. Visfatin, also known as nicotinamide phosphoribosyltransferase, is produced by visceral adipose tissue and immune cells and may influence glucose metabolism, oxidative stress and inflammatory signalling [19]. Although the exact clinical value of these adipokines is still evolving, their altered levels may reflect the metabolic and inflammatory background of NAFLD [10,19].

Routine liver enzymes and ultrasonography are useful in clinical evaluation, but they do not fully reflect adipose tissue dysfunction, insulin resistance or inflammatory burden [2,3]. Therefore, studying adipokine profiles may provide additional insight into the biological mechanisms that connect visceral obesity with hepatic steatosis. This is particularly relevant in Indian and South Asian populations, where central adiposity and insulin resistance may occur even at relatively lower body mass index levels [20]. The present study was undertaken to evaluate serum adipokine profiles in patients with NAFLD and compare them with healthy controls. The study also aimed to examine the association of adiponectin, leptin, resistin and visfatin with anthropometric and biochemical parameters, including body mass index, waist circumference, fasting glucose, lipid profile, liver enzymes and insulin resistance.

## Materials and methods

Study design

This case-control study was designed to assess the serum adipokine profile among patients with non-alcoholic fatty liver disease (NAFLD) and to compare the findings with apparently healthy individuals. The study was conducted in a tertiary care teaching hospital, with coordinated involvement of the Departments of Biochemistry, General Medicine and Radiology. Patients who attended the outpatient or inpatient services with clinical, biochemical, or radiological suspicion of fatty liver disease were screened for eligibility. Those who were diagnosed with NAFLD on abdominal ultrasonography and fulfilled the selection criteria were included as cases. Age- and sex-matched individuals without clinical or ultrasonographic evidence of fatty liver disease were enrolled as controls.

Study setting

The study was carried out at Chalmeda AnandRao Institute of Medical Sciences, Karimnagar. Patient recruitment was done through the Department of General Medicine, while ultrasonographic confirmation of fatty liver was provided by the Department of Radiology. Biochemical investigations and adipokine estimation were performed in the Department of Biochemistry. The institutional facilities allowed systematic clinical assessment, radiological diagnosis, routine biochemical testing and specialised adipokine analysis within the same hospital setting.

Study duration

The study was conducted over a period of 16 months, from January 2025 to April 2026. During this period, eligible NAFLD patients and healthy controls were recruited after applying the predefined inclusion and exclusion criteria. Clinical evaluation, anthropometric measurements, blood sample collection, routine biochemical testing, insulin resistance assessment and adipokine estimation were completed within the study period.

Study population

A total of 100 participants were included in the study. The case group consisted of 50 patients diagnosed with NAFLD, while the control group included 50 apparently healthy adults matched for age and sex. NAFLD cases were selected after confirming fatty liver on abdominal ultrasonography and excluding significant alcohol intake and other recognised secondary causes of hepatic steatosis. Controls were selected from healthy volunteers who had no evidence of fatty liver on ultrasonography, normal liver function tests and no major metabolic or systemic illness.

Sample size and calculation

The sample size consisted of 50 cases and 50 controls. The sample size was calculated for comparison of mean adipokine levels between two independent groups, namely NAFLD patients and healthy controls. Based on earlier studies reporting differences in serum adiponectin levels between NAFLD cases and controls, an expected mean difference of 2.5 µg/mL with a pooled standard deviation of 4.0 µg/mL was considered for calculation. Using a 5% level of significance, 80% power and the formula for comparison of two independent means, the minimum required sample size was approximately 41 participants in each group.

After allowing for possible incomplete records, sample rejection, or non-response, the sample size was increased by about 10%. Therefore, a minimum of 45 participants per group was required. For better feasibility and balanced group comparison, the final sample size was rounded to 50 NAFLD patients and 50 healthy controls, giving a total sample size of 100 participants. This number was considered suitable for the present study based on the availability of eligible participants during the study period, feasibility of specialised adipokine testing and findings from previous studies that reported measurable differences in adipokine levels between NAFLD patients and healthy controls. A total sample of 100 participants allowed comparison between the two groups while maintaining practical feasibility for laboratory-based adipokine analysis.

Inclusion criteria

Patients aged 18 to 60 years were included in the NAFLD group if they had ultrasonographic evidence of fatty liver and were willing to provide written informed consent. For the control group, apparently healthy adults in the same age range were included if they had no clinical, biochemical, or ultrasonographic evidence of fatty liver disease. Participants were enrolled only after their eligibility was confirmed by clinical history, routine biochemical evaluation and radiological findings.

Exclusion criteria

Participants were excluded if they had a history of significant alcohol consumption, viral hepatitis, autoimmune liver disease, drug-induced liver injury, chronic kidney disease, malignancy, pregnancy, or acute systemic illness. Patients receiving corticosteroids, hepatotoxic drugs, lipid-lowering medications, insulin-sensitising agents, or any drugs known to affect hepatic fat accumulation or adipokine levels were also excluded. Individuals with incomplete clinical records, inadequate fasting samples, or unsuitable blood specimens were not included in the final analysis.

Diagnosis of NAFLD

NAFLD was diagnosed on the basis of abdominal ultrasonographic evidence of hepatic steatosis, after excluding significant alcohol intake and other secondary causes of fatty liver, as recommended in current clinical guidance for the evaluation of NAFLD/MASLD [2,3]. Ultrasonographic features suggestive of hepatic steatosis included increased hepatic echogenicity compared with the renal cortex, poor visualisation of intrahepatic vascular margins and reduced visibility of the diaphragm in more marked steatosis. The ultrasound examination was performed by an experienced radiologist who was blinded to the biochemical and adipokine findings. When applicable, fatty liver was graded as mild, moderate, or severe according to the degree of hepatic echogenicity and associated sonographic changes [3].

Ultrasonographic assessment and grading

Abdominal ultrasonography was used to confirm hepatic steatosis and to grade fatty liver severity. All ultrasound examinations were performed by an experienced radiologist with expertise in abdominal imaging. The radiologist was blinded to the biochemical parameters, insulin resistance status and adipokine results of the participants at the time of examination. Fatty liver was identified based on increased hepatic echogenicity compared with the renal cortex, reduced clarity of intrahepatic vascular margins and decreased visualisation of the diaphragm in more advanced cases. Based on these sonographic findings, fatty liver was graded as mild, moderate, or severe. This approach helped maintain uniformity in radiological assessment and reduced observer-related bias.

Clinical and anthropometric assessment

All participants underwent detailed clinical evaluation. Information was collected regarding age, sex, dietary pattern, physical activity, history of diabetes mellitus, hypertension, dyslipidaemia, medication use, alcohol intake and other relevant medical history. Anthropometric measurements were recorded using standard methods. Body weight was measured in kilograms using a calibrated weighing scale, and height was measured in centimetres using a stadiometer. Body mass index was calculated by dividing weight in kilograms by height in metres squared. Waist circumference was measured midway between the lower margin of the last rib and the iliac crest at the end of normal expiration. Blood pressure was recorded in the sitting position after adequate rest.

Blood sample collection

After an overnight fast of 8 to 12 hours, approximately 5 mL of venous blood was collected from each participant under aseptic precautions. Blood samples were collected in appropriate tubes depending on the required investigation. Fluoride-containing tubes were used for glucose estimation, while plain tubes were used for serum separation. Serum was separated by centrifugation at 3,000 rpm for 10 minutes. Routine biochemical parameters were analysed using freshly separated serum wherever possible. For adipokine estimation, serum aliquots were stored at -20°C until analysis. Repeated freezing and thawing of samples was avoided to maintain sample integrity.

Biochemical analysis

Fasting blood glucose, total cholesterol, triglycerides, high-density lipoprotein cholesterol, low-density lipoprotein cholesterol, aspartate aminotransferase, alanine aminotransferase, alkaline phosphatase, total bilirubin, albumin and other routine biochemical parameters were measured using standard laboratory methods on an automated biochemistry analyser. Very-low-density lipoprotein cholesterol was calculated from triglyceride values wherever applicable. All biochemical tests were performed according to the manufacturer’s instructions, and internal quality control procedures were followed throughout the analysis.

Estimation of insulin resistance

Fasting insulin was estimated using a standard immunoassay method. Insulin resistance was assessed using the homeostatic model assessment of insulin resistance, commonly expressed as Homeostatic Model Assessment of Insulin Resistance (HOMA-IR). The following formula was used:

HOMA-IR = fasting insulin (µIU/mL) × fasting glucose (mg/dL) / 405

Higher HOMA-IR values were interpreted as indicating greater insulin resistance. This parameter was used to examine the association of adipokines with metabolic dysfunction among patients with NAFLD.

Adipokine estimation

Serum adiponectin, leptin, resistin and visfatin levels were measured using commercially available human enzyme-linked immunosorbent assay (ELISA) kits. Human adiponectin was estimated using Human Adiponectin ELISA Kit, Abcam, Cambridge, UK, Cat. No. ab99968. Human leptin was measured using Human Leptin ELISA Kit, Abcam, Cambridge, UK, Cat. No. ab179884. Human resistin was estimated using Human Resistin ELISA Kit, Abcam, Cambridge, UK, Cat. No. ab183364. Human visfatin was measured using Human Visfatin ELISA Kit, Abcam, Cambridge, UK, Cat. No. ab267658. These kits are sandwich ELISA-based assays suitable for quantitative estimation of the respective adipokines in human serum or plasma samples.

All assays were performed in the Department of Biochemistry by trained laboratory personnel according to the manufacturer’s instructions. Fasting serum samples were used after appropriate storage and controlled thawing. Standards, controls and participant samples were added to antibody-coated microplate wells, followed by incubation, washing, enzyme conjugate addition, substrate reaction and optical density reading using an ELISA plate reader at the recommended wavelength. The concentration of each adipokine was calculated from the standard calibration curve. Adiponectin values were expressed in µg/mL, whereas leptin, resistin and visfatin values were expressed in ng/mL. Samples were analysed in duplicate wherever feasible, and kit controls were included in each run to maintain analytical reliability.

Quality control

All laboratory procedures were carried out using standard operating protocols. Internal quality control sera were used for routine biochemical investigations, and analyser calibration was performed as required. For ELISA-based adipokine assays, the validity of each run was confirmed using kit controls and standard curves. Samples with haemolysis, lipemia, inadequate volume, or improper storage conditions were excluded from analysis. Laboratory personnel involved in adipokine estimation were blinded to the case or control status of the participants to minimise measurement bias.

Study variables

The primary variables of the study were serum adiponectin, leptin, resistin and visfatin levels. Secondary variables included body mass index, waist circumference, fasting blood glucose, fasting insulin, HOMA-IR, lipid profile parameters and liver function tests. The relationship between adipokines and anthropometric or biochemical markers was analysed to understand their association with obesity, insulin resistance, dyslipidaemia and hepatic dysfunction in NAFLD.

Ethical considerations

The study was carried out after approval from the Institutional Ethics Committee of Chalmeda AnandRao Institute of Medical Sciences, Karimnagar, India, with approval/reference number CAIMS/IEC/DRAFT/2024/128, dated: December 18, 2024. Written informed consent was obtained from all participants before enrolment. The study purpose, blood sample collection procedure, expected benefits and confidentiality measures were explained to each participant in a language they could understand. Participation was completely voluntary, and participants were free to withdraw at any stage without any effect on their routine medical care. Personal details were removed during data entry and analysis to maintain confidentiality.

Statistical analysis

Data were first entered in Microsoft Excel (Microsoft Corporation, Redmond, Washington, United States) and then analysed using IBM SPSS Statistics for Windows, Version 26.0 (IBM Corp., Armonk, New York, United States). Continuous variables were summarised as mean with standard deviation, and categorical variables were expressed as frequency and percentage. The independent Student’s t-test was applied to compare normally distributed continuous variables between NAFLD patients and controls. Categorical variables were compared using the chi-square test. Correlations between serum adipokines and anthropometric or biochemical parameters were assessed using Pearson’s correlation coefficient for normally distributed variables and Spearman’s correlation coefficient for variables that were not normally distributed. A p-value of less than 0.05 was considered statistically significant.

## Results

Distribution of the study population

The study included 100 participants, with 50 patients diagnosed with non-alcoholic fatty liver disease (NAFLD) and 50 healthy controls. The two groups were comparable for age and sex distribution. The mean age of the NAFLD group was 43.8 ± 9.6 years, while the mean age of the control group was 41.9 ± 8.8 years. The difference was not statistically significant (p = 0.304). Males constituted 58.0% of the NAFLD group and 52.0% of the control group (χ² = 0.36, p = 0.548), showing that both groups were reasonably matched for sex distribution (Table 1).

**Table 1:** Demographic profile of the study participants Independent Student’s t-test was used for comparison of age between the NAFLD and control groups. Chi-square test was used for comparison of categorical variables such as sex, family history of diabetes and hypertension. NAFLD: non-alcoholic fatty liver disease.

Variable	NAFLD group (n = 50)	Control group (n = 50)	Test value	p-value
Age, years	43.8 ± 9.6	41.9 ± 8.8	t = 1.03	0.304
Male sex	29 (58.0%)	26 (52.0%)	χ² = 0.36	0.548
Female sex	21 (42.0%)	24 (48.0%)	χ² = 0.36	0.548
Family history of diabetes	18 (36.0%)	9 (18.0%)	χ² = 4.11	0.043
Hypertension	16 (32.0%)	6 (12.0%)	χ² = 5.83	0.016

Anthropometric and clinical parameters

Patients with NAFLD had significantly higher body mass index, waist circumference and systolic blood pressure than controls. The mean body mass index was 29.1 ± 3.8 kg/m² in the NAFLD group compared with 23.7 ± 2.9 kg/m² among controls (p < 0.001) (Table 2). Waist circumference was also higher in NAFLD patients (96.4 ± 8.7 cm) than in controls (82.6 ± 7.9 cm; p < 0.001). These findings indicate a strong association between NAFLD and central obesity.

**Table 2:** Anthropometric and clinical parameters in NAFLD patients and controls Independent Student’s t-test was used to compare continuous variables between the NAFLD and control groups. NAFLD: non-alcoholic fatty liver disease.

Parameter	NAFLD group (n = 50)	Control group (n = 50)	t-value	p-value
Body mass index, kg/m²	29.1 ± 3.8	23.7 ± 2.9	7.98	<0.001
Waist circumference, cm	96.4 ± 8.7	82.6 ± 7.9	8.31	<0.001
Systolic blood pressure, mmHg	128.6 ± 12.4	118.2 ± 10.8	4.47	<0.001
Diastolic blood pressure, mmHg	82.4 ± 7.6	76.8 ± 6.9	3.86	<0.001

Biochemical and metabolic profile

The NAFLD group showed a clear adverse metabolic profile compared with controls. Fasting blood glucose was significantly higher in NAFLD patients (112.8 ± 18.5 mg/dL) than in controls (91.6 ± 10.7 mg/dL; p < 0.001). Fasting insulin and HOMA-IR were also markedly increased in NAFLD patients, suggesting greater insulin resistance (Table 3).

**Table 3:** Biochemical profile of the study groups Independent Student’s t-test was used to compare biochemical parameters between the NAFLD and control groups. NAFLD: non-alcoholic fatty liver disease, HOMA-IR: Homeostatic Model Assessment of Insulin Resistance, HDL-C: high-density lipoprotein cholesterol, LDL-C: low-density lipoprotein cholesterol, ALT: alanine aminotransferase, AST: aspartate aminotransferase.

Parameter	Reference range	NAFLD group (n = 50)	Control group (n = 50)	t-value	p-value
Fasting blood glucose, mg/dL	70-100	112.8 ± 18.5	91.6 ± 10.7	7.01	<0.001
Fasting insulin, µIU/mL	2-25	13.6 ± 4.8	7.5 ± 2.9	7.69	<0.001
HOMA-IR	<2.5	3.8 ± 1.2	1.7 ± 0.6	11.07	<0.001
Total cholesterol, mg/dL	<200	198.4 ± 36.7	166.2 ± 29.5	4.84	<0.001
Triglycerides, mg/dL	<150	186.5 ± 42.3	124.7 ± 31.8	8.25	<0.001
HDL-C, mg/dL	>40 in men; >50 in women	39.8 ± 7.4	48.6 ± 8.1	5.67	<0.001
LDL-C, mg/dL	<100	121.3 ± 30.5	92.7 ± 24.8	5.15	<0.001
ALT, U/L	7-56	56.8 ± 21.4	28.6 ± 10.2	8.42	<0.001
AST, U/L	10-40	44.7 ± 16.9	25.4 ± 8.7	7.18	<0.001

Serum triglycerides and low-density lipoprotein cholesterol were significantly elevated, while high-density lipoprotein cholesterol was lower in the NAFLD group. Liver enzymes, particularly alanine aminotransferase and aspartate aminotransferase, were significantly higher among NAFLD patients.

Serum adipokine profile

A distinct adipokine imbalance was observed in patients with NAFLD. Serum adiponectin was significantly lower in the NAFLD group (5.9 ± 2.1 µg/mL) than in controls (10.8 ± 3.4 µg/mL; p < 0.001) (Table 4).

**Table 4:** Comparison of serum adipokine levels between NAFLD patients and controls Independent Student’s t-test was used to compare serum adipokine levels between the NAFLD and control groups. NAFLD: non-alcoholic fatty liver disease.

Adipokine	Reference range	NAFLD group (n = 50)	Control group (n = 50)	t-value	p-value
Adiponectin, µg/mL	5-30 µg/mL	5.9 ± 2.1	10.8 ± 3.4	8.67	<0.001
Leptin, ng/mL	Men: 0.5-13.8; Women: 1.1-27.6 ng/mL	24.6 ± 8.9	12.3 ± 5.4	8.35	<0.001
Resistin, ng/mL	4-16 ng/mL	14.8 ± 4.6	8.7 ± 3.1	7.79	<0.001
Visfatin, ng/mL	10-60 ng/mL	32.5 ± 10.2	18.9 ± 7.6	7.56	<0.001

In contrast, pro-inflammatory adipokines were significantly elevated in NAFLD patients. Mean leptin level was 24.6 ± 8.9 ng/mL in the NAFLD group compared with 12.3 ± 5.4 ng/mL in controls. Resistin and visfatin were also significantly higher in NAFLD patients, with all comparisons showing p < 0.001. These findings suggest that NAFLD is associated with reduced protective adipokine activity and increased inflammatory adipokine expression.

Association of adipokines with metabolic parameters

Correlation analysis among NAFLD patients showed that adiponectin had a significant negative correlation with body mass index, waist circumference, triglycerides, HOMA-IR and alanine aminotransferase. The strongest negative association was observed with HOMA-IR (r = -0.58, p < 0.001). Leptin showed positive correlations with body mass index (r = 0.61, p < 0.001), waist circumference (r = 0.56, p < 0.001) and HOMA-IR (r = 0.54, p < 0.001) (Table 5).

**Table 5:** Correlation of adipokines with selected clinical and biochemical parameters in NAFLD patients Pearson’s correlation coefficient was used to assess the correlation between adipokine levels and selected clinical and biochemical parameters. A p-value <0.05 was considered statistically significant. NAFLD: non-alcoholic fatty liver disease, HOMA-IR: Homeostatic Model Assessment of Insulin Resistance, ALT: alanine aminotransferase.

Parameter	Adiponectin r (p-value)	Leptin r (p-value)	Resistin r (p-value)	Visfatin r (p-value)
Body mass index	-0.52 (<0.001)	0.61 (<0.001)	0.43 (0.002)	0.38 (0.006)
Waist circumference	-0.49 (<0.001)	0.56 (<0.001)	0.39 (0.005)	0.35 (0.012)
Triglycerides	-0.46 (0.001)	0.42 (0.003)	0.36 (0.010)	0.33 (0.019)
HOMA-IR	-0.58 (<0.001)	0.54 (<0.001)	0.47 (0.001)	0.45 (0.001)
ALT	-0.39 (0.005)	0.34 (0.016)	0.41 (0.003)	0.37 (0.008)

Resistin and visfatin also showed positive correlations with insulin resistance and liver enzyme levels, indicating their possible relationship with metabolic stress and hepatic injury in NAFLD.

Ultrasonographic grading of fatty liver

Among the 50 NAFLD patients, 21 patients (42.0%) had grade I fatty liver, 19 patients (38.0%) had grade II fatty liver and 10 patients (20.0%) had grade III fatty liver. With increasing ultrasound grade, there was a progressive rise in body mass index, triglycerides, HOMA-IR, leptin, resistin and visfatin levels, while adiponectin showed a declining trend. The difference across grades was statistically significant for adiponectin, leptin and HOMA-IR (Table 6).

**Table 6:** Adipokine and metabolic profile according to ultrasonographic grade of NAFLD One-way analysis of variance (ANOVA) was used to compare BMI, HOMA-IR and serum adipokine levels across Grade I, Grade II and Grade III NAFLD groups. A p-value <0.05 was considered statistically significant. NAFLD: non-alcoholic fatty liver disease, HOMA-IR: Homeostatic Model Assessment of Insulin Resistance.

Parameter	Grade I (n = 21)	Grade II (n = 19)	Grade III (n = 10)	F-value	p-value
BMI, kg/m²	27.6 ± 3.1	29.4 ± 3.5	31.5 ± 4.2	4.28	0.019
HOMA-IR	3.1 ± 0.9	3.9 ± 1.0	4.9 ± 1.3	10.46	<0.001
Adiponectin, µg/mL	6.8 ± 2.0	5.7 ± 1.8	4.3 ± 1.5	6.18	0.004
Leptin, ng/mL	20.8 ± 7.2	25.1 ± 8.0	31.4 ± 9.6	5.71	0.006
Resistin, ng/mL	12.9 ± 3.8	15.2 ± 4.1	17.8 ± 5.2	4.39	0.018
Visfatin, ng/mL	28.1 ± 8.4	33.5 ± 9.2	39.4 ± 11.6	5.08	0.010

## Discussion

The present study evaluated serum adipokine levels in patients with NAFLD and compared them with age- and sex-matched healthy controls. The main finding was an altered adipokine pattern in NAFLD, with lower adiponectin and higher leptin, resistin and visfatin levels. These changes were associated with higher body mass index, increased waist circumference, adverse lipid profile, raised liver enzymes and greater insulin resistance. These observations support the concept that NAFLD is closely linked with metabolic dysfunction and adipose tissue activity, rather than being only a hepatic fat accumulation disorder [2,3]. However, as this was an observational case-control study, these findings should be interpreted as associations and not as proof of a direct causal role of adipokines in disease pathogenesis.

Patients with NAFLD had significantly higher body mass index and waist circumference than controls. This finding is important because central adiposity is closely related to hepatic fat deposition. Visceral adipose tissue increases free fatty acid flux to the liver, which may promote intrahepatic triglyceride accumulation and metabolic stress [6,8]. These processes may further contribute to oxidative injury, lipotoxicity and progression of fatty liver disease [8,9]. The higher frequency of hypertension and family history of diabetes in the NAFLD group also indicates the clustering of cardiometabolic risk factors. This is consistent with the current understanding that fatty liver disease forms part of a wider metabolic disorder rather than an isolated liver abnormality [1,2].

The NAFLD group also showed higher fasting glucose, fasting insulin and Homeostatic Model Assessment of Insulin Resistance (HOMA-IR) values, reflecting greater insulin resistance. Insulin resistance is an important metabolic abnormality in NAFLD because it increases adipose tissue lipolysis, hepatic de novo lipogenesis and impaired fatty acid oxidation [8,9]. In the present study, patients with NAFLD also had higher triglycerides and low-density lipoprotein cholesterol (LDL-C), along with lower high-density lipoprotein cholesterol (HDL-C). This lipid pattern is commonly seen in metabolic syndrome and may increase cardiovascular risk [2,6]. This has clinical relevance because cardiovascular disease is one of the major causes of morbidity and mortality in patients with fatty liver disease [6,7].

A major observation in this study was the reduction in serum adiponectin levels among patients with NAFLD. Adiponectin has insulin-sensitising, anti-inflammatory and lipid oxidation-enhancing effects [12,13]. Lower adiponectin levels may therefore be linked with insulin resistance, lipid accumulation and hepatic inflammation. Similar findings have been reported in earlier studies, where reduced adiponectin was observed in NAFLD, especially in patients with obesity and metabolic abnormalities [14,15]. In the present study, adiponectin showed a negative correlation with body mass index, waist circumference, triglycerides, HOMA-IR and alanine aminotransferase (ALT). This suggests that lower adiponectin may reflect increasing metabolic burden and liver enzyme elevation, although a causal relationship cannot be confirmed from the present design.

Serum leptin levels were significantly higher in patients with NAFLD. Leptin normally regulates appetite and energy balance, but in obesity, chronic hyperleptinaemia may coexist with leptin resistance [13,16]. Under such conditions, leptin may be associated with inflammatory signalling and fibrogenic pathways in the liver [16,17]. In this study, leptin showed positive correlations with body mass index, waist circumference and HOMA-IR, indicating that increased leptin was closely related to adiposity and insulin resistance. These findings are in agreement with previous reports linking elevated leptin levels with NAFLD and metabolic severity [16]. Still, the present results should be viewed as supportive evidence of association rather than direct evidence of leptin-mediated hepatic injury.

Resistin levels were also higher in the NAFLD group than in controls. Resistin has been linked with insulin resistance, endothelial dysfunction and inflammatory activity [18]. In the present study, resistin showed positive correlations with body mass index, triglycerides, HOMA-IR and ALT. This may indicate that resistin is associated with both metabolic load and hepatic biochemical changes in NAFLD. However, the biological role of resistin in humans is not fully consistent across studies, as circulating levels may be affected by adiposity, inflammation and immune cell activity. Therefore, resistin may be more useful as part of a wider metabolic and inflammatory profile rather than as an independent diagnostic marker.

Visfatin was also significantly increased in patients with NAFLD. Visfatin is secreted by visceral adipose tissue and immune cells and has been associated with glucose metabolism, oxidative stress and inflammation [19]. Its positive correlation with HOMA-IR, triglycerides, ALT and obesity-related indices suggests that visfatin may reflect the metabolic and inflammatory background of NAFLD. However, compared with adiponectin and leptin, the evidence for visfatin in NAFLD is less uniform [10,19]. Hence, visfatin should be interpreted as a supportive marker of metabolic inflammation and not as a stand-alone diagnostic or prognostic indicator.

When NAFLD patients were analysed according to ultrasonographic grade, higher grades of fatty liver were associated with higher body mass index, HOMA-IR, leptin, resistin and visfatin levels, while adiponectin showed a gradual decline. This pattern suggests that increasing sonographic severity of hepatic steatosis was accompanied by greater insulin resistance and adipokine imbalance. At the same time, the limitation of ultrasound-based grading must be acknowledged. Ultrasonography is widely available and useful for routine detection of fatty liver, but it is less sensitive for mild steatosis and cannot reliably assess steatohepatitis or fibrosis [2,3]. Therefore, the present ultrasound findings should be interpreted as an assessment of hepatic steatosis rather than a complete measure of histological disease severity.

The findings may have particular relevance in the Indian population. South Asians are known to develop central obesity, insulin resistance and type 2 diabetes at relatively lower body mass index levels [20]. As a result, NAFLD may occur even in individuals who are not markedly obese by conventional criteria. In the present study, waist circumference and insulin resistance showed clear associations with adipokine alterations, suggesting that visceral adiposity may be an important contributor to the metabolic profile seen in NAFLD in this population.

The study has some strengths, including a clearly defined case-control design, inclusion of matched healthy controls and simultaneous assessment of multiple adipokines along with anthropometric, biochemical and insulin resistance parameters. However, some limitations should be considered. The diagnosis and grading of NAFLD were based on ultrasonography rather than liver biopsy or magnetic resonance-based fat quantification. The study was conducted at a single centre with a modest sample size, which may limit generalisability. The observational design also prevents any conclusion regarding causality. Inflammatory cytokines and fibrosis markers were not assessed, which could have provided additional information on disease activity and progression. Larger multicentre prospective studies using more sensitive imaging, histological correlation where feasible, and broader inflammatory and fibrosis markers are needed to confirm these findings.

Limitations

The study has a few limitations. As this was a case-control study, it could show associations but could not establish a cause-and-effect relationship between adipokine changes and NAFLD. The sample size was modest, and the study was conducted at a single centre, which may limit the wider applicability of the findings. NAFLD was diagnosed using ultrasonography, which is practical and routinely used, but it is less sensitive for mild steatosis and cannot reliably assess steatohepatitis or fibrosis when compared with liver biopsy or magnetic resonance-based methods. In addition, inflammatory cytokines and fibrosis markers were not included, which could have provided more information on disease activity and progression.

Despite these limitations, the study objectives were clearly addressed. The findings showed a consistent adipokine imbalance in NAFLD, with lower adiponectin and higher leptin, resistin and visfatin levels. These changes were associated with obesity indices, dyslipidaemia, insulin resistance and raised liver enzymes, supporting the role of adipose tissue dysfunction in metabolic-associated fatty liver disease. Larger multicentre and longitudinal studies are needed to confirm these findings and to assess whether adipokines can be useful for predicting disease progression or monitoring response to treatment.

## Conclusions

The present study found that patients with non-alcoholic fatty liver disease showed altered serum adipokine levels compared with healthy controls. Adiponectin levels were lower, while leptin, resistin and visfatin levels were higher in the NAFLD group. These changes were associated with higher body mass index, increased waist circumference, dyslipidaemia, insulin resistance and raised liver enzymes. The findings indicate that adipokine alterations may reflect the metabolic disturbances seen in NAFLD. However, because this was a single-centre case-control study, these results should be interpreted as associations. Larger prospective studies are needed to confirm whether adipokines have value as supportive markers for disease assessment or follow-up in NAFLD.
